# Supplementary material for: Single-cell spatial transcriptomics reveals molecular patterns of selective neuronal vulnerability to α-synuclein pathology in a transgenic mouse model of Lewy body disease
Source: bioRxiv. 2024 Aug 1:2024.07.31.606032. Preprint. [Version 1] doi: 10.1101/2024.07.31.606032 (PMC11451628; doi:10.1101/2024.07.31.606032)
Supplement: Supplementary file 1 [file media-1.pdf]

Supplementary figures for: *Single-cell spatial transcriptomics reveals molecular patterns of selective neuronal vulnerability to α-synuclein pathology in a transgenic mouse model of Lewy body disease*

Liam Horan-Portelance[1], Michiyo Iba[1], Dominic J. Acri[2], J. Raphael Gibbs[3], Eliezer Masliah[1,4], Mark R. Cookson[2*]

[1] Molecular Neuropathology Unit, Laboratory of Neurogenetics, National Institute on Aging, National Institutes of Health, Bethesda, MD 20892, USA.
[2] Cell Biology and Gene Expression Section, Laboratory of Neurogenetics, National Institute on Aging, National Institutes of Health, Bethesda, MD 20892, USA.
[3] Computational Biology Group, Laboratory of Neurogenetics, National Institute on Aging, National Institutes of Health, Bethesda, MD 20892, USA.
[4] Division of Neuroscience, National Institute on Aging, National Institutes of Health, Bethesda, MD 20892, USA.

* Corresponding author: Mark R. Cookson, PhD, Laboratory of Neurogenetics, NIA, NIH, Building 35, Room 1A116, MSC3707 35, Convent Drive, Bethesda, MD, 20892-3707, USA. Phone +1-301-451-3870. Email: cookson@mail.nih.gov

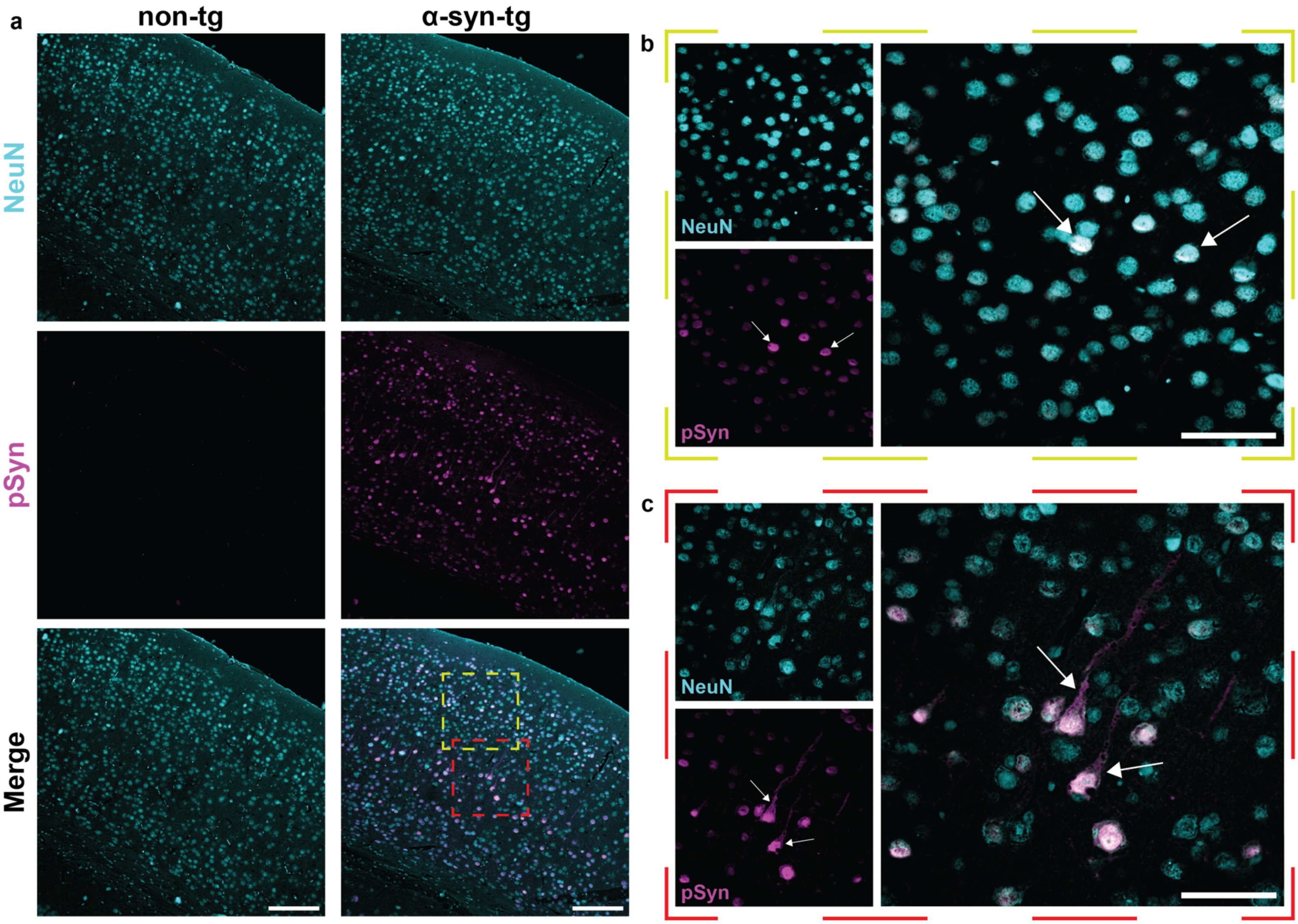

Supplementary Fig. 1: Representative images of pSyn pathology in non-tg and α-syn-tg brains. Following the Xenium run, sections were stained for NeuN (neuronal marker) and α-syn phosphorylated at Ser129 (pSyn). **a** Representative overview (10x magnification) images of non-tg (left) and α-syn-tg (right) cortices, showing staining of pan-neuronal marker NeuN (top, cyan), pSyn (middle, magenta), and merged. Images display absence of $pSyn^+$ cells in the non-tg cortex, and abundant neuronal pSyn staining in the α-syn-tg brain. Scale bar = 150 µm. **b** Representative high-magnification (40x) image of outer-layer (layer 2/3) neurons from an α-syn-tg cortex, showing neurons stained with NeuN (top left, cyan) and pSyn (bottom left, magenta), and merged (right). pSyn staining patterns highlight the nuclear localization of pathology in these cells (arrows). Scale bar = 50 µm. **c** Representative high-magnification (40x) image of deep-layer (layer 5) neurons from an α-syn-tg cortex, showing neurons stained with NeuN (top left, cyan) and pSyn (bottom left, magenta), and merged (right). pSyn staining patterns highlight the severe pathology in these cells extending out of the nucleus, to the cytoplasm and axons (arrows). Scale bar = 50 µm.

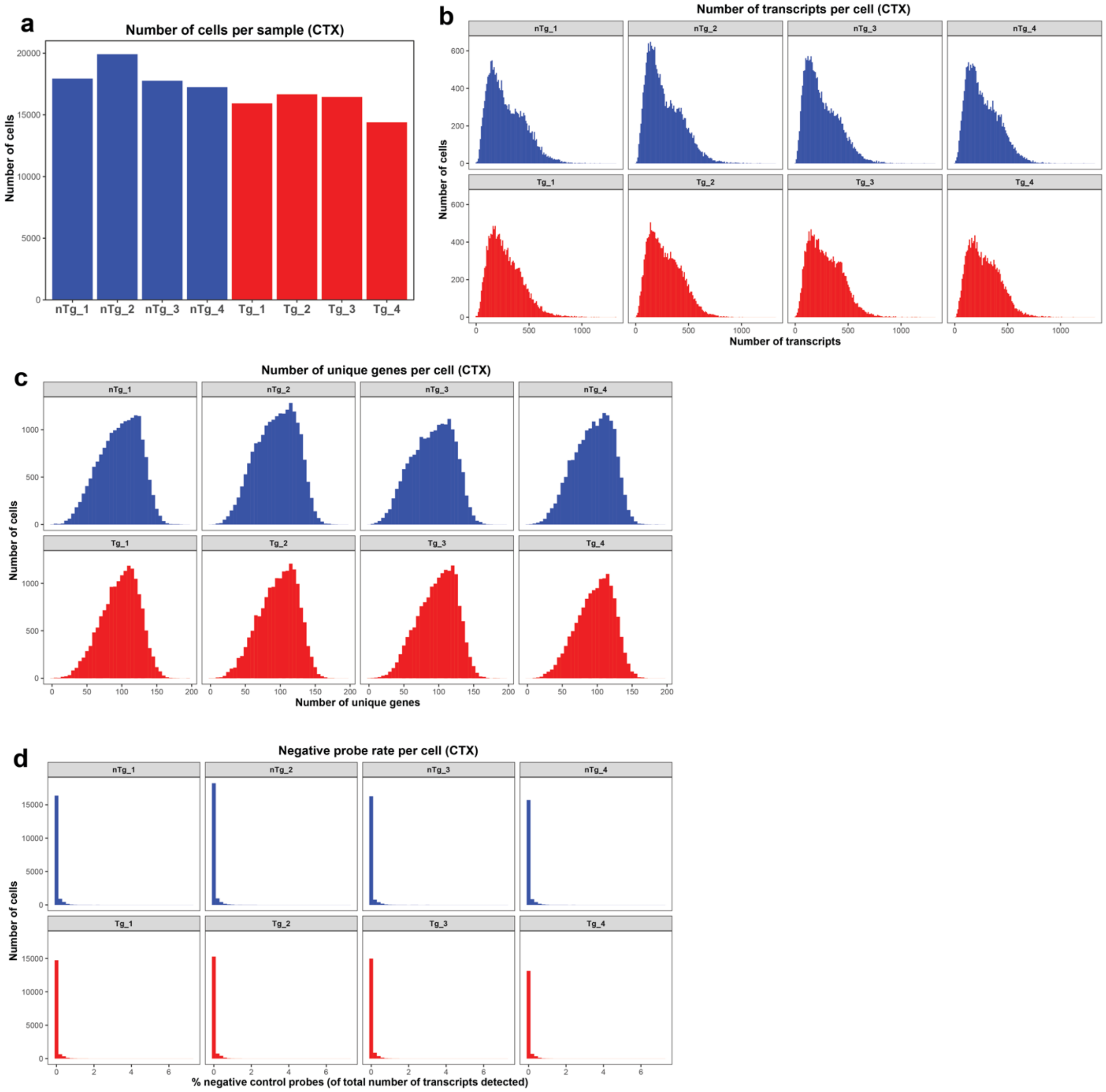

Supplementary Fig. 2: Xenium quality control measures for cortical cells. a Number of cells detected in each sample. **b** Number of transcripts detected per cell in each sample. **c** Number of unique genes detected per cell in each sample. **d** Negative probe rate for each cell in each sample, calculated as the number of detected negative control probes divided by the total number of detected probes in a given cell.

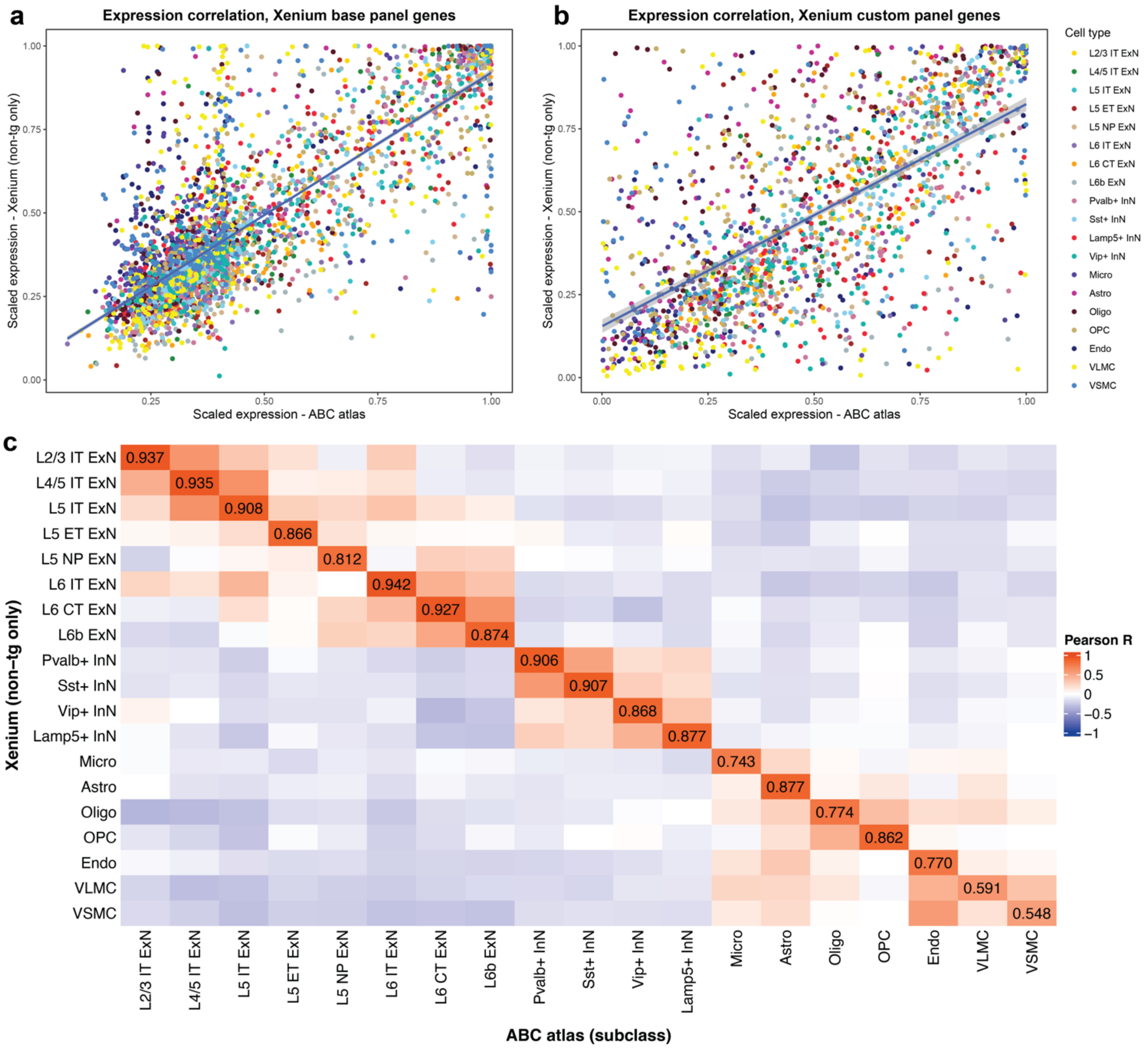

Supplementary Fig. 3: Gene expression and cell type agreement between sequencing-based and imaging-based transcriptomics. a-b Correlation of expression of the 248 genes in the Xenium base panel **(a)** and the 99 genes in our custom panel (excluding *hSNCA*) **(b)** between our Xenium data and the Allen Brain Cell atlas scRNA-seq data. Expression for each gene was scaled across cell types to allow for direct comparison, and each point represents scaled expression of a single gene in a single cell type (see Methods, "Comparison of Xenium to single-cell RNA sequencing data"). **c** Heatmap showing Pearson correlation of expression profiles between cell types, in Xenium and the ABC atlas, considering only the Xenium base panel genes. Scaled gene expression profiles (i.e., scaled expression of every gene in the base panel) for each cell type across both technologies were compared, and a Pearson coefficient was calculated between every cell type (see Methods, "Comparison of Xenium to single-cell RNA sequencing data"). Pearson R is displayed for each matching cell type pair.

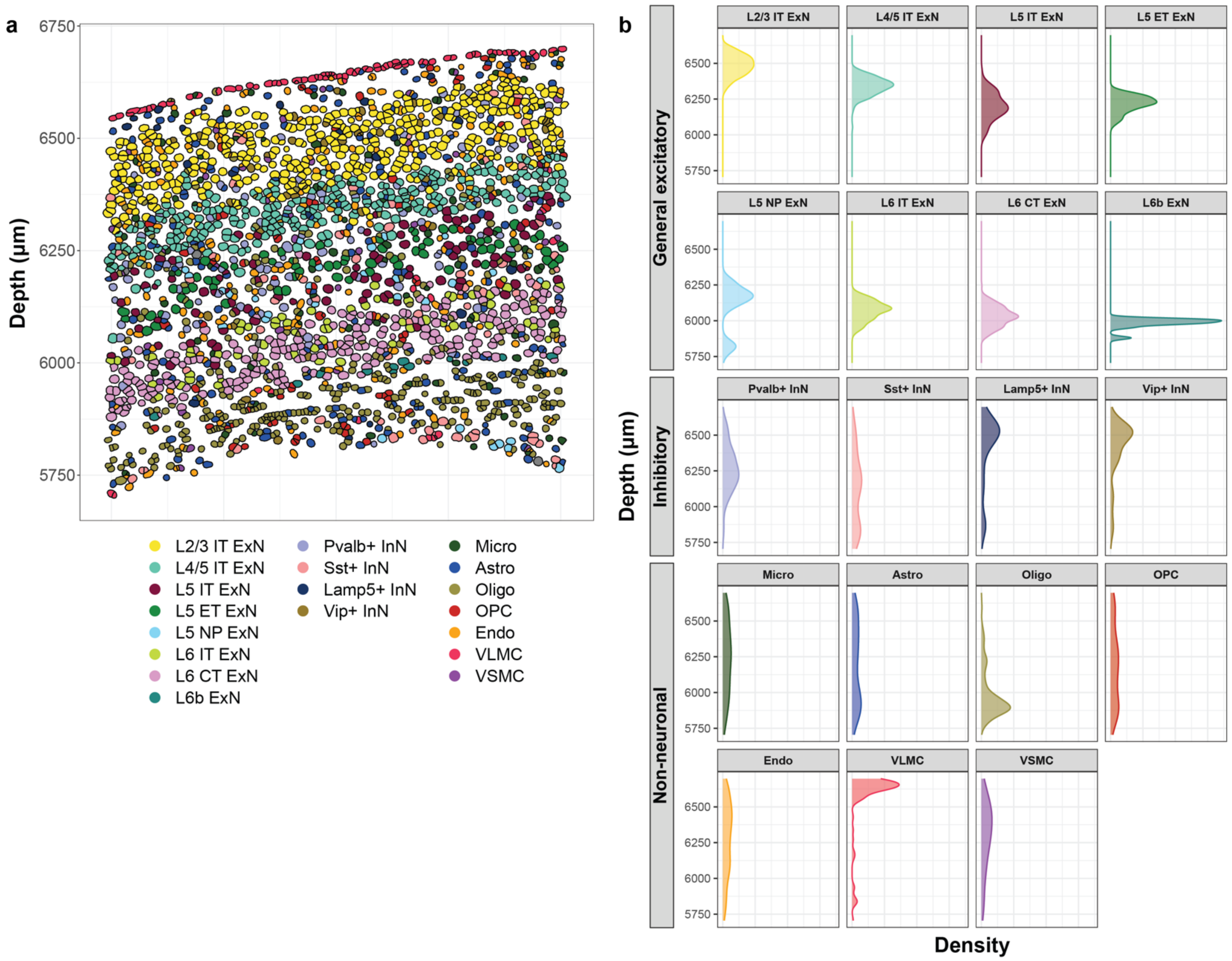

Supplementary Fig. 4: Spatial information ground truth further validates Xenium cell type assignments. a Representative image containing all 6 cortical layers and the corpus collosum (same as seen in Fig. 1b). Each dot represents a cell, colored by cell type. Depth is indicated to the left, represented as the x-coordinate (in μm). **b** Density plots showing distribution of each of the cell types as a function of depth through the cortex in the representative FOV in **(a)**.

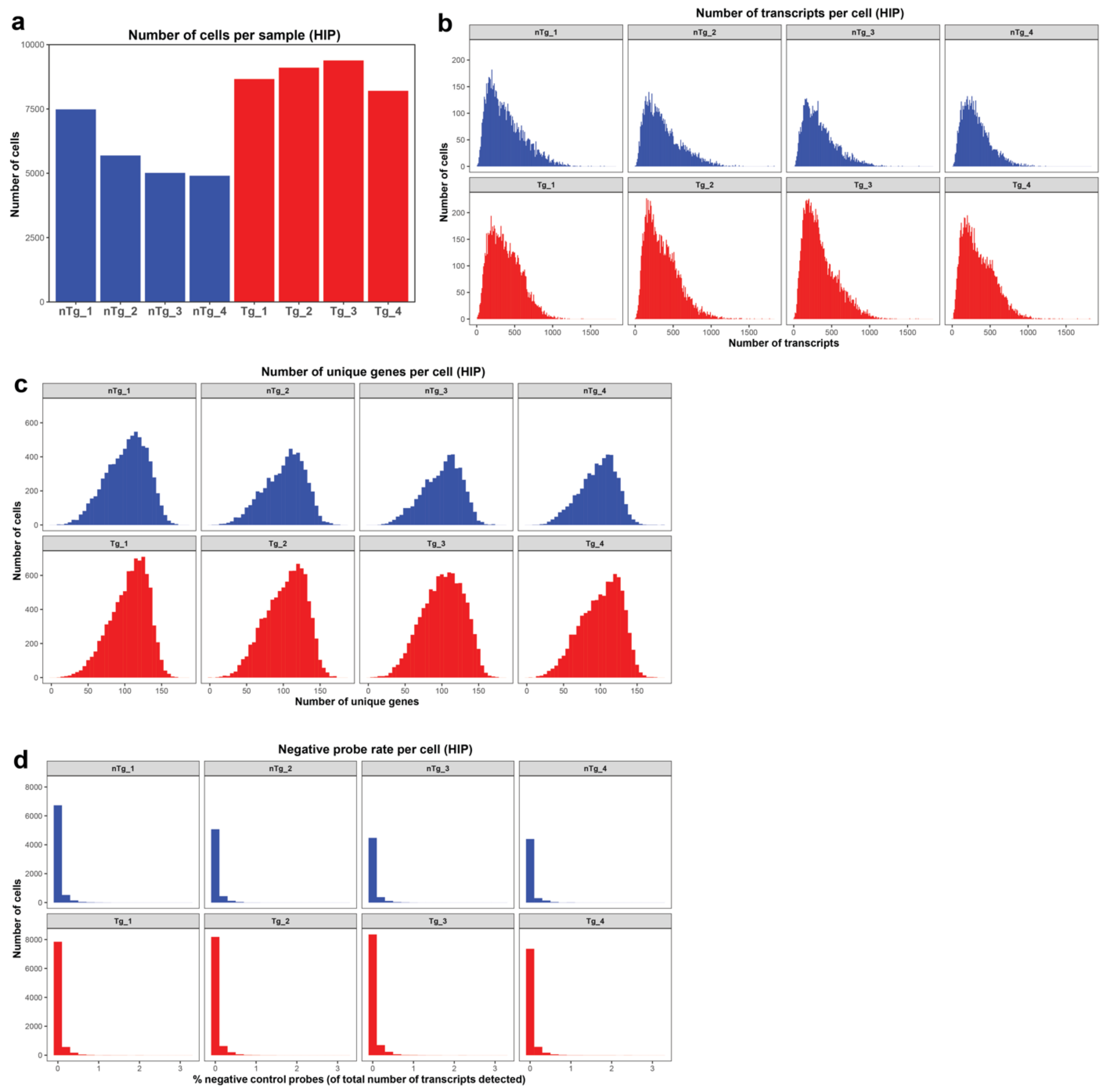

Supplementary Fig. 5: Xenium quality control measures for hippocampal cells. a Number of cells detected in each sample. **b** Number of transcripts detected per cell in each sample. **c** Number of unique genes detected per cell in each sample. **d** Negative probe rate for each cell in each sample, calculated as the number of detected negative control probes divided by the total number of detected probes in a given cell.

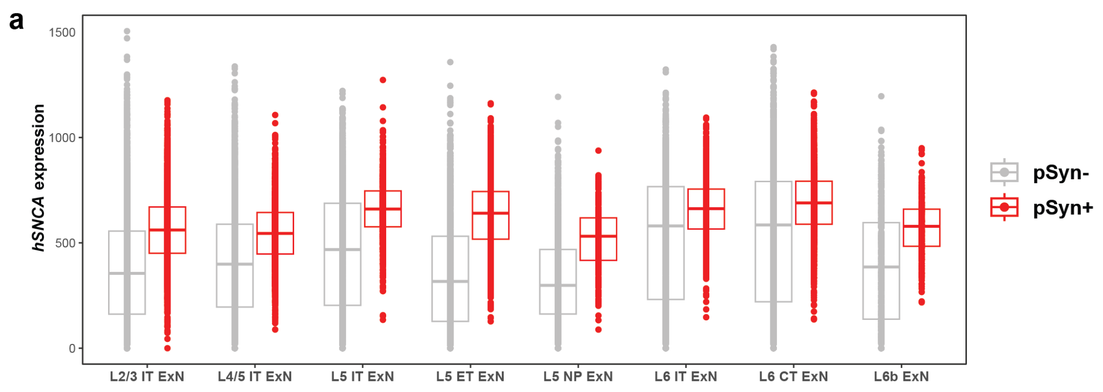

Supplementary Fig. 6: *hSNCA* expression by individual cortical cells. a *hSNCA* expression at the single-cell level, by cell type and split by pSyn status (grey = pSyn$^-$, red = pSyn$^+$). pSyn$^+$ cells demonstrate slightly higher *hSNCA* expression than pSyn$^-$ cells across cell types, on average. Each dot represents 1 cell, and expression = expm1(log-normalized counts).

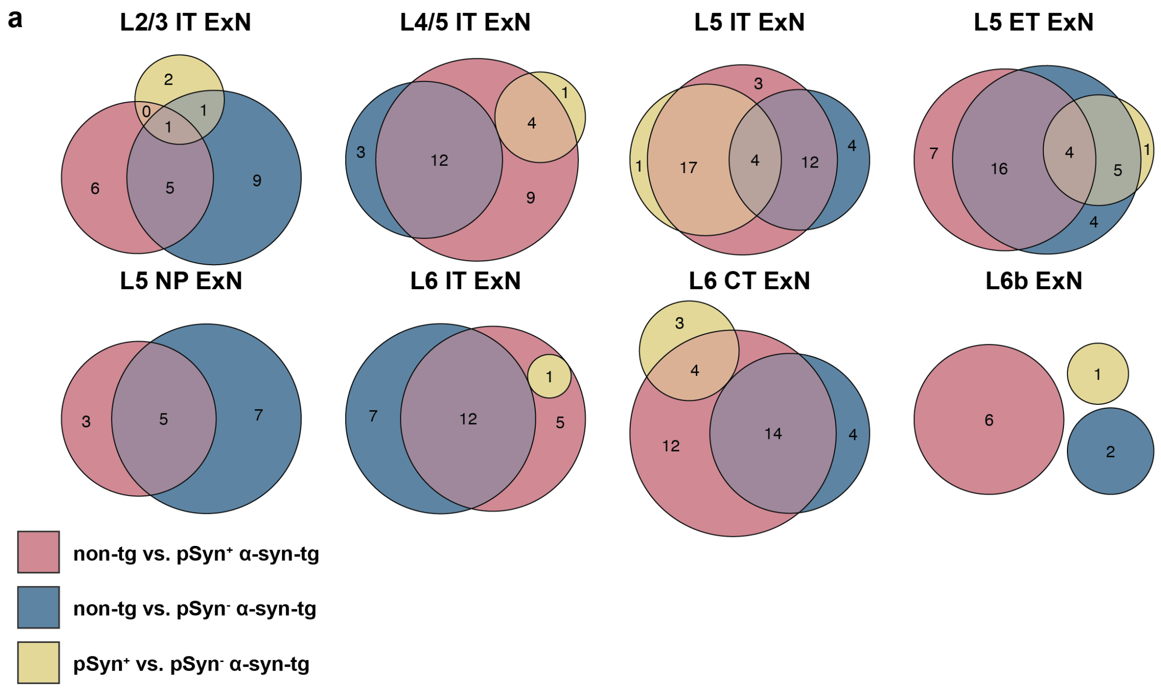

Supplementary Fig. 7: DEG overlap across cell types between pseudo-bulk comparisons. a Euler plots for all general ExN subtypes showing the overlap of DEGs between the 3 differential expression comparisons (red = non-tg vs. pSyn^{+} α-syn-tg; blue = non-tg vs. pSyn^{-} α-syn-tg; yellow = pSyn^{+} vs. pSyn^{-} α-syn-tg). Size of the circles is scaled to the number of genes in each group, which is denoted by the numbers inside the circles.
